# A comparative study of MRI-induced RF heating in pediatric and adult populations with epicardial and endocardial implantable electronic devices

**DOI:** 10.1109/EMBC48229.2022.9871087

**Published:** 2022-07

**Authors:** Fuchang Jiang, Bhumi Bhusal, Pia Sanpitak, Gregory Webster, Andrada Popescu, Daniel Kim, Giorgio Bonmassar, Laleh Golestanirad

**Affiliations:** Department of Biomedical Engineering, Northwestern University, Evanston, IL 60608 USA.; Department of Radiology, Northwestern University, Chicago, IL 60611 USA.; Department of Radiology, Northwestern University, Chicago, IL 60611 USA.; Division of Cardiology, Ann and Robert H. Lurie Children’s Hospital of Chicago, Northwestern University Feinberg School of Medicine, 225 East Chicago Avenue, Box 21, Chicago, IL, 60611, USA; Division of Medical Imaging, Ann & Robert H. Lurie Children’s Hospital of Chicago, Chicago, IL, USA; Department of Radiology, Northwestern University, Chicago, IL 60611 USA.; A. A. Martinos Center for Biomedical Imaging, Massachusetts General Hospital, Boston, MA, USA; Department of Radiology and Department of Biomedical Engineering, Northwestern University, Chicago, IL 60611 USA.

## Abstract

Patients with congenital heart defects, inherited arrhythmia syndromes, and congenital disorders of cardiac conduction often receive a cardiac implantable electronic device (CIED). At least 75% of patients with CIEDs will need magnetic resonance imaging (MRI) during their lifetime. In 2011, the US Food and Drug Administration approved the first MR-conditional CIEDs for patients with endocardial systems, in which leads are passed through the vein and affixed to the endocardium. The majority of children, however, receive an epicardial CIED, where leads are directly sewn to the epicardium. Unfortunately, an epicardial CIED is a relative contraindication to MRI due to the unknown risk of RF heating. In this work, we performed anthropomorphic phantom experiments to investigate differences in RF heating between endocardial and epicardial leads in both pediatric and adult-sized phantoms, where adult endocardial CIED was the control.

## I. Introduction

Cardiac implantable electronic devices (CIEDs) treat cardiac conduction disorders, replacing sinus and atrioventricular nodal function and providing high voltage defibrillation for abnormal heart rhythms in both adults and children [1, 2]. Two types of CIED leads are commonly used in clinical practice depending on patient's body size and venous anatomy: Adults and older children typically receive endocardial systems, with the leads inserted directly through the subclavian vein and the implantable pulse generator (IPG) placed in the left or right subpectoral pocket [3, 4]. Infants and young children often receive an epicardial system, which requires a sternotomy or thoracotomy to sew the cardiac lead directly to the myocardium. The IPG is then usually placed inferior to the abdominal rectus. Although cases of young children being implanted with the endocardial system are rare, there are reports of young patients (< 8 kg) with a transvenous pacing system [4]. Also, for adult patients with residual right-to-left shunts, or those with chambers that cannot be accessed by the transvenous route, epicardial leads implantation is necessary [5].

At least 75% of patients with CIEDs will require magnetic resonance imaging (MRI) exams during their lifetime [6]. Unfortunately, however, MRI is not readily accessible to patients with electronic implants due to risks associated with the interaction of MRI fields and the implanted device. Since the first report of such harmful effects in 1989 [7] significant improvement has been made in implant design and manufacturing, leading to the first generation of MR-conditional CIEDs that obtained FDA approval in 2011. Such improvements include for example, reduction of ferromagnetic material to reduce the risk of device dislodgement, and enhanced device programming to reduce the risk of unintended tissue stimulation. Tissue heating from radiofrequency (RF) excitation fields, however, remains a major issue. This “antenna effect” happens when the electric field of the MRI transmit coil couples with an elongated conductive lead, causing the specific absorption rate (SAR) of the RF energy to amplify at the lead’s tip [8-10]. Consequently, patients with such leads are either contraindicated to receive MRI, or only approved to undergo MRI under strictly controlled conditions.

The Heart and Rhythm Society expert consensus statement on MRI in patients with CIEDs provides Class I recommendation supported by Level A evidence (i.e., strong recommendation supported by high-quality evidence) that patients with MR-conditional CIEDs should only undergo MRI when the product labeling is adhered to [11]. Because FDA-approved MR-conditional labeling is currently only available for CIEDs with endocardial leads, patients with epicardial devices have been mostly excluded from MRI exams [12]. This restriction primarily occurs in the vulnerable pediatric population, or in adults who have congenital heart disease, as the majority of epicardial CIEDs are implanted in children.

The goal of this study was to compare MRI-induced RF heating at tips of epicardial and endocardial leads with realistic trajectories implanted in adult and pediatric phantoms during MRI at 1.5 T. To our knowledge, such comparative study has not been performed before. Because body size and lead trajectory are two factors that substantially affect RF heating [8, 13-15], we performed experiments in human-shaped phantoms of different sizes implanted with leads that followed patient-derived realistic trajectories. We provide the first preliminary results on RF heating of epicardial leads in clinically relevant populations.

## II. Methods

### A. Phantom Design and Construction

**Figure 1.**
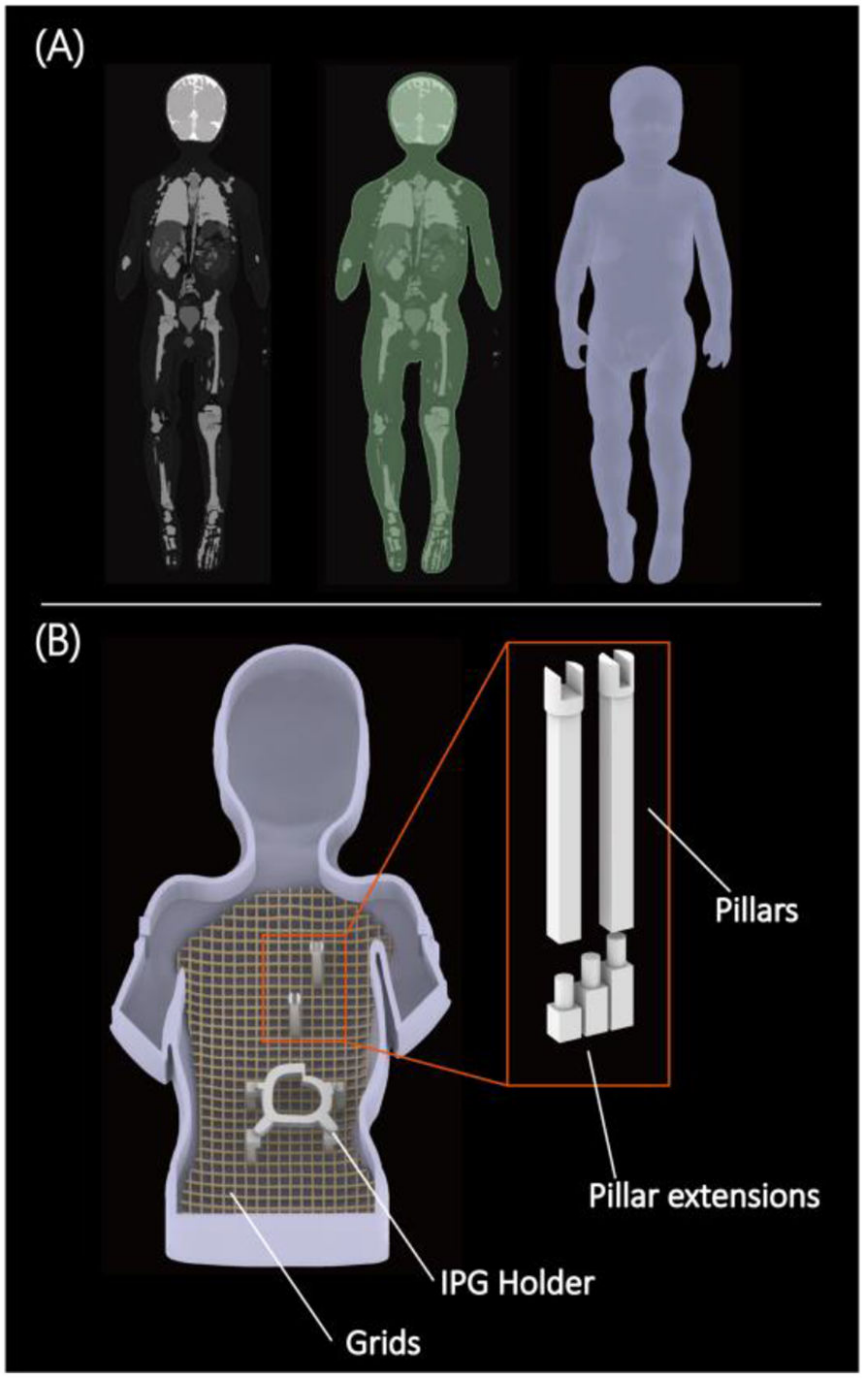
(A) MRI of a 29-month-old child was used to create 3D models of the child's silhouette. (B) Head and torso phantom with girds, pillars, and different height of pillar extensions to adjust the positioning of the CIEDs.

Experiments were performed in two human-shaped head-and-torso phantoms representing an adult (length 65 cm, width 45 cm, volume 26 L, weight equivalent value 150lb) and a 29-month-old child (length 53 cm, width 30 cm, volume 11 L, weight equivalent value 30lb). Design and construction of the adult phantom is described elsewhere [16]. The pediatric phantom was designed and 3D printed based on segment MR images of a 29-month-old child [17]. 3D slicer (Slicer 4.10, http://slicer.org) was used to smooth segmented MRI masks and create body-shaped surface models [18]. Surface models were further processed in a CAD tool (Rhino 6.0, Robert McNeel & Associates for Windows, Seattle, WA) to create 3D printable objects. We also designed grids, posts, and IPG holders to help positioning CIEDs in well-controlled clinically relevant configurations (Figure 1).

**Figure 2.**
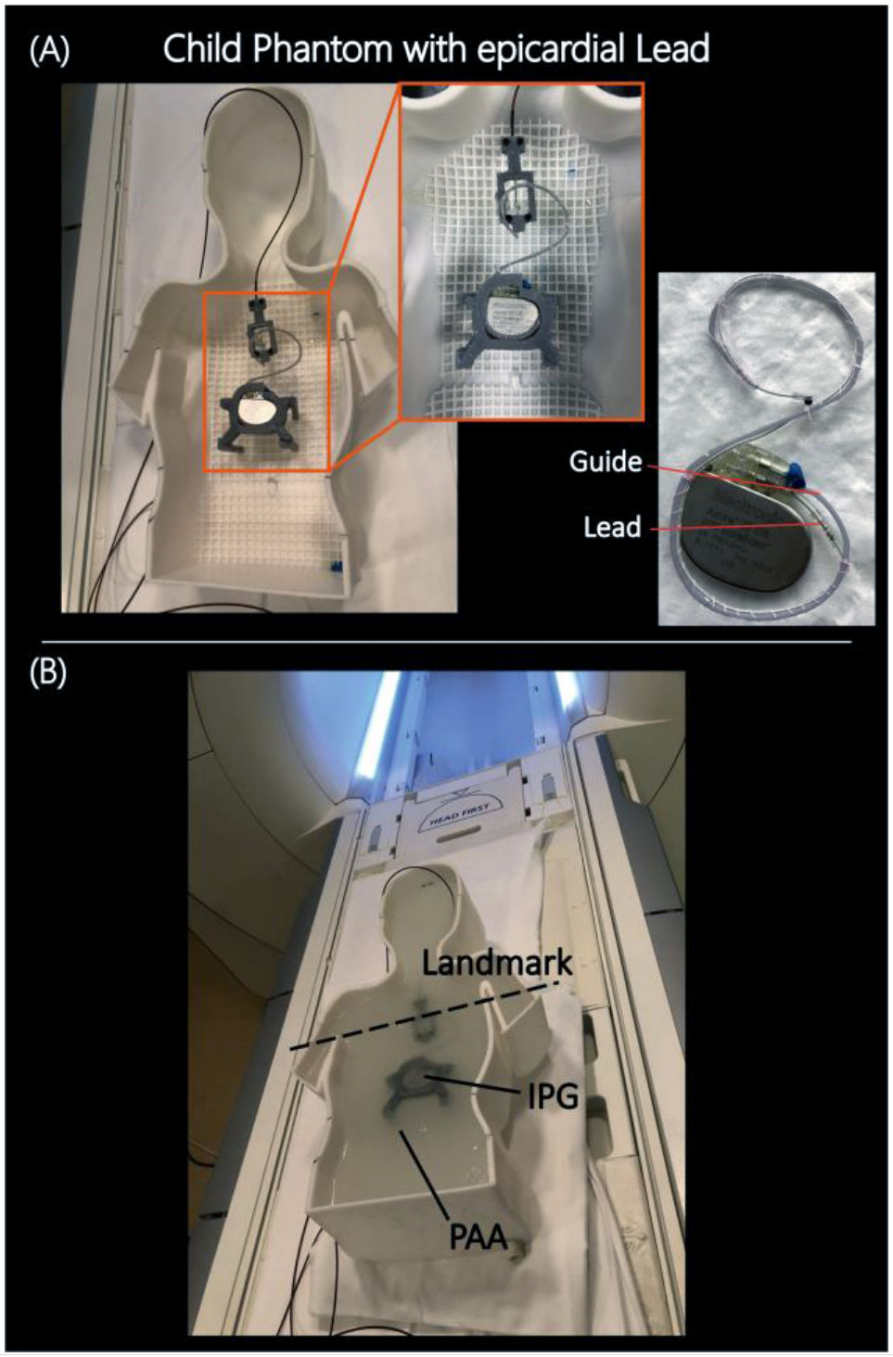
(A) Assembled phantom with CIED and temperature measuring setup before pouring into PAA. 3D printed trajectory guide was attached to the lead (B) Assembled phantom with CIED and temperature measuring setup after pouring into PAA. Chest imaging landmark was used during MR scans.

Phantoms were filled with polyacrylamide (PAA) with conductivity of *σ* = 0.47 *S*/*m* and relative permittivity of *ε*_*r*_ = 88 mimicking average body tissue. We used 9 L and 22 L of PAA to fill the pediatric and adult phantoms, respectively, such that the leads and the IPG were fully submerged at 3 cm depth beneath the surface of the gel (Figure 2).

### B. CIED Configurations

**Figure 3.**
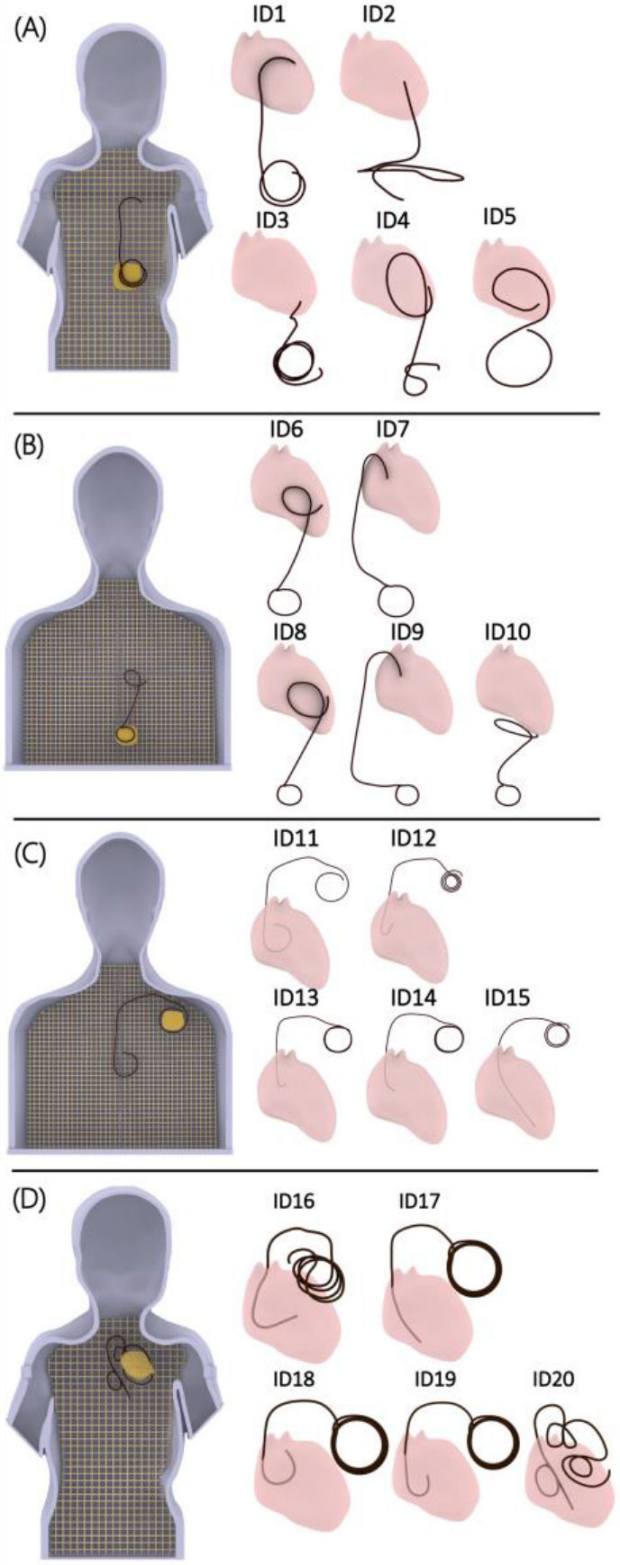
Twenty distinct lead trajectories were investigated, including: (A) 5 epicardial leads in the child phantom, (B) 5 epicardial leads in the adult phantom, (C) 5 endocardial leads in the adult phantom, and (D) 5 endocardial leads in the child phantom.

Experiments with the epicardial CIED were performed with a 35 cm lead (Medtronic CapSure^®^ EPI 4965) connected to a Medtronic Azure^™^ XT DR MRI SureScan pulse generator placed in the phantom’s abdomen. Experiments with endocardial leads were performed with a 58 cm lead (CapSurFix Novus MRI^™^ SureScan^™^ bipolar 5076) connected to the same pulse generator placed in the left infraclavicular region. For each phantom (e.g., child vs. adult), we modeled 5 clinically relevant lead trajectories based on either computed tomography (CT) images of our patients, X-ray images of patients available in the literature, or expert opinion of a pediatric electrophysiologist who implants CIEDs in children and adults (Figure 3).

**Figure 4.**
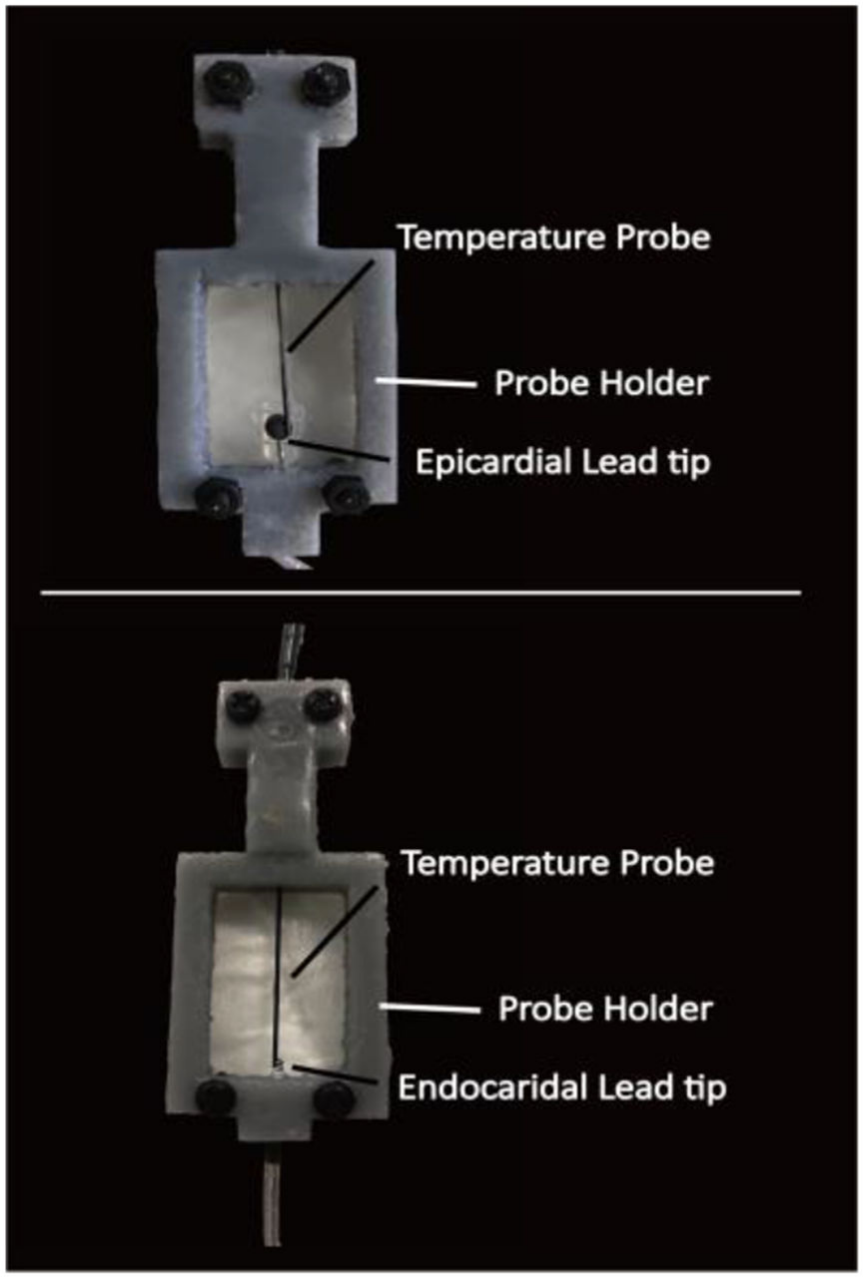
Close view of contact between temperature probe and epicardial/endocardial lead tip.

Temperature measurement was performed using MR-compatible fiber optic probes (OSENSA, Vancouver BC, Canada, resolution 0.01 °C) secured at lead tips. To ensure good thermal contact during experiments, a home-made holder was designed that securely held the probe in contact with the tip of the lead as illustrated in Figure 4.

Measurements were performed in a 1.5 T Siemens Aera scanner (Siemens Healthineers, Erlangen, Germany) with phantoms positioned with the chest at the isocenter. A high-SAR T_1_-weighted turbo spin echo (T_1_-TSE) sequence was used for experiments (TE=7.3 ms, TR= 897 ms, B_1_+ = 4.9 μT, Acquisition time= 280 s).

## III. Results

**Figure 5.**
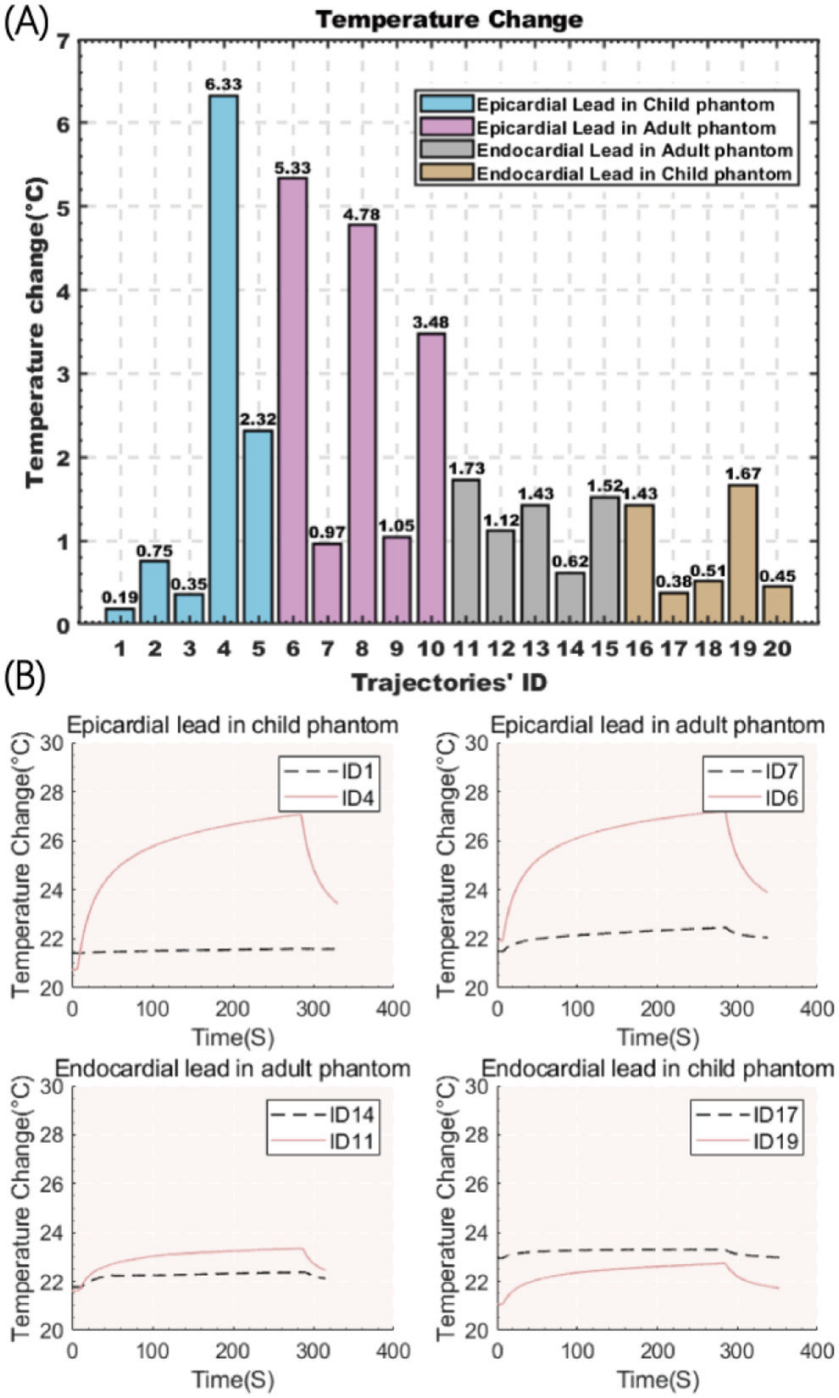
(A) Temperature rise at tips of the lead with 20 different trajectories showed in Figure 3. (B) maximum and minimum of the exponential RF heating measurement for each group

Figure 5 gives the magnitude of RF heating recorded at the tip of each lead after 280 s RF exposure. For each group, the temporal profile of RF heating is also given for cases that generated maximum and minimum heating.

The mean ± standard deviation of RF heating was 1.99±2.57 °C and 3.12±2.04 °C in pediatric and adult phantoms with the epicardial leads, respectively, and 0.89±0.61 °C and 1.28±0.43 °C in pediatric and adult phantoms with endocardial leads. The highest RF heating was observed in the pediatric phantom with an epicardial lead. Both groups are normally distributed as calculated by the Anderson-Darling normality test (P>0.05). A one-tail independent t-test at significant level of *α* = 0.05 showed that epicardial leads generated significantly higher heating compared to endocardial leads (Pvalue = 0.037).

Overall, we observed higher variation in the RF heating of epicardial leads compared to the variation of RF heating of endocardial leads. However, heating is trajectory-dependent as seen in figure 5. This could be explained by the fact that in endocardial CIED implantation, the IPG is consistently placed in the subpectoral pocket and leads are passed through the subclavian vein, which limits the variation in their trajectory. In contrast, there are no rules or guidelines for placing epicardial systems. As a result, surgeons position the IPG and leads based on prior experience and best practices handed from surgeon-to-surgeon, leading to substantial variation in lead trajectories. Because RF heating of an elongated implant highly depends on its trajectory which in turn determines the degree of coupling of the lead with MRI electric fields [19-22], the larger variation in epicardial lead trajectory translates to larger variation in RF heating.

This work did not have enough power to draw inferences as to the effect of body size (i.e., child vs adult) on RF heating. More work is required to establish the safety of MRI in age-specific pediatric populations with epicardiac leads.

## Clinical Relevance—

This work provides a quantitative comparison of MRI RF heating of epicardial and endocardial leads in pediatric and adult populations.
